# Role of Migratory Birds in Spreading Crimean-Congo Hemorrhagic Fever, Turkey

**DOI:** 10.3201/eid2008.131547

**Published:** 2014-08

**Authors:** Hakan Leblebicioglu, Cafer Eroglu, Kiraz Erciyas-Yavuz, Murat Hokelek, Mustafa Acici, Hava Yilmaz

**Affiliations:** Ondokuz Mayis University Medical School, Samsun, Turkey (H. Leblebicioglu, C. Eroglu, M. Hokelek, H. Yilmaz);; Ondokuz Mayis University Ornithology Research Center, Samsun (K. Erciyas-Yavuz);; Ondokuz Mayis University Faculty of Veterinary Medicine, Samsun (M. Acici)

**Keywords:** Crimean-Congo hemorrhagic fever, CCHF, birds, Turkey, epidemiology, migration, ticks

## Abstract

We investigated migratory birds’ role in spreading Crimean-Congo hemorrhagic fever virus (CCHFV) through attached ticks. We detected CCHFV RNA in ticks on migratory birds in Turkey. Two isolates showed similarity with CCHFV genotype 4, suggesting a role for ticks in CCHFV epidemics in Turkey and spread of CCHFV by birds.

Crimean-Congo hemorrhagic fever (CCHF), an illness characterized by fever and hemorrhage, is caused by CCHF virus (CCHFV) (family *Bunyaviridae*, genus *Nairovirus*). CCHFV has been isolated from many species of ticks, primarily *Hyalomma* spp (*1*). In Turkey, CCHFV has been detected mostly in *Hyalomma* spp. ticks (*2*). Although CCHF is common in Turkey, Iran, Pakistan, and Afghanistan, sporadic cases are reported from the neighboring countries and the Balkans (*1*). No case was reported before 2002 from Turkey, but the annual number of cases increased exponentially until 2009. A total of 7,192 CCHF cases were reported during 2002–2012 to the Ministry of Health (Turkish Ministry of Health, unpub. data).

CCHF is encountered in the inner parts of the Black Sea and Middle Anatolia regions, which provide a suitable climate for *Hyalomma* spp. ticks. Infected ticks carrying the virus might have been transported to Turkey on migratory birds. Turkey is a land bridge on this primary migration route for many migratory birds breeding in the Palearctic and wintering in Africa (*3*).

The role of infected ticks carried on migratory birds has not been investigated as a cause for increased CCHF in Turkey. Our aim was to investigate the role of the migratory birds in spreading CCHFV through attached ticks.

## The Study

Birds were caught by mist-nets, banded (ringed), and examined for ticks at the Cernek Bird Ringing Station (41°36’N, 36°05’E) in the Kizilirmak Delta in Turkey, an internationally important wetland area for birds (*4*). We conducted the study during the spring and autumn migration seasons in 2010 and 2011 and in spring 2012. Bird species and number of ticks on each species were recorded. Each tick was speciated by examining morphologic characteristics under stereomicroscope (*5*).

The identified ticks were placed in tubes with steel beads and homogenized at the maximum speed (50 Hz) for 10 min in TissueLyser LT device (QIAGEN, Hilden, Germany). RNA was isolated according to the manufacturer’s recommendations by using High Pure Viral Nucleic Acid Kit (Roche Applied Science, Mannheim, Germany), but as a small modification, the homogenized tick mixture was kept at 37°C for 1 h.

In accordance with the manufacturer’s recommendations, we obtained viral cDNA using the RevertAid First Strand cDNA Synthesis Kit (Thermo Scientific, Vilnius, Lithuania). Real-time PCR was performed by using the combination of primer pairs and the FastStart TaqMan Probe Master Kit (Roche Applied Science), as described by Yapar et al. (*6*), for each tick sample. cDNA from patient samples, which previously had been determined as positive, were used as the positive control sample.

We performed conventional PCR only on positive samples obtained from real-time PCR. CCHFV small (S) segment (encoding for the nucleocapsid protein) specific primer pairs (F3: 3′-GAATGTGCATGGGTTAGCTC-5′ and R2: 3′-GACATCACAATTTCACCAGG-5′) and same PCR conditions defined by Schwarz et al. (*7*) were used in the PCR. Sequence analysis was performed on the ≈260-bp PCR product, when positivity was detected, by using the primers of F3 and R2 in the ABI 310 Genetic Analyzer (Applied Biosystems, Foster City, CA, USA).

Sequences organized by using Chromas Lite Program (http://technelysium.com.au) were entered in GenBank. Our sequences and GenBank sequences were aligned in MEGA 5.1 (http://www.megasoftware.net), and the phylogenetic tree was drawn on the basis of the 260 bp of the S segment of the CCHFV genome. To compare the sequences and phylogenetic analysis, we used the maximum-likelihood method.

We found attached ticks on 65 (0.5%) of the 13,377 captured and banded birds, which represented 17 species. A total of 188 ticks collected on these birds belonged to *Ixodes*, *Hyalomma*, *Haemaphysalis*, and *Rhipicephalus* genera (Table). Only 2 ticks (*Hyalomma* sp. and *Ixodes* sp.) were CCHF positive by PCR.

**Table:** Ticks collected on migratory birds in a study of the role of migration in spreading Crimean-Congo hemorrhagic fever, Turkey

Bird (species)	No. infested/no. captured (%)	Mean Intensity*	Tick characteristic†					
			Species	Age, no.			Sex, no.	
				Larvae	Nymph		F	M
Common blackbird (*Turdus merula*)	17/514 (3.3)	6.8	*Ixodes hexagonus*				3	
			*I. ricinus*				6	
			*Ixodes* spp.	9	95			
			*Rhipicephalus bursa*					1
			*Haemaphysalis* spp.	2				
Song thrush (*T. philomelos*)	2/238 (0.8)	1	*Ixodes* spp.		2			
Thrush nightingale (*Luscinia luscinia*)	1/150 (0.7)	1	*Hyalomma* sp.				1	
Common redstart (*Phoenicurus phoenicurus*)	3/457 (0.7)	1.7	*I. ricinus*				1	
			*Ixodes* sp.		1			
			*Hyalomma* spp.		3			
European robin (*Erithacus rubecula*)	16/3,106 (0.5)	1.4	*I. hexagonus*				2	
			*I. ricinus*				2	1
			*Ixodes* spp.‡	1	16			
European pied flycatcher (*Ficedula hypoleuca*)	1/58 (1.7)	11	*Hyalomma* spp.		11			
Common chaffinch (*Fringilla coelebs*)	2/194 (1.0)	1	*I. hexagonus*				1	
			*Ixodes* sp.		1			
Dunnock (*Prunella modularis*)	1/41 (2.4)	1	*Ixodes* sp.		1			
Eurasian blackcap (*Sylvia atricapilla*)	8/1,478 (0.5)	1	*I. hexagonus*				1	
			*I. ricinus*					1
			*Ixodes* spp.	1	5			
Garden warbler (*S. borin*)	2/1,183 (0.2)	1	*Ixodes* spp.		2			
Lesser whitethroat (*S. curruca*)	1/154 (0.7)	1	*Ixodes* sp.	1				
Common chiffchaff (*Phylloscopus collybita)*	1/1,104 (0.1)	2	*Ixodes* spp.	2				
*Great reed warbler (Acrocephalus arundinaceus)*	3/30 (10.0)	2.7	*Hyalomma* spp.‡		6		2	
Savi’s warbler (*Locustella luscinioides*)	2/20 (10.0)	1	*I. ricinus*				1	
			*Ixodes* sp.		1			
Cetti’s warbler (*Cettia cetti*)	3/187 (1.6)	1	*Ixodes* spp.		3			
Eurasian blue tit (*Cyanistes caeruleus*)	1/10 (10.0)	1	*I. hexagonus*				1	
Red-backed shrike (*Lanius collurio*)	1/193 (0.5)	1	*Hyalomma* sp.		1			
Total	65/9,117 (0.7)			16	148		21	3

*Mean overall intensity of infestation per bird species. †Blank cells indicate no data. ‡One tick was CCHFV-positive by PCR.

CCHFV (Samsun T3–2010 and Samsun T18–2010) was detected in *Hyalomma* spp. (nymphs) (21 [4.8%] ticks) and *Ixodes* spp. (nymphs) (127 [0.8%]) on great reed warblers (*Acrocephalus arundinaceus*) (9.7% of banded birds) and on European robins (*Erithacus rubecula*) (0.5% of banded birds), respectively.

**Figure 1:**
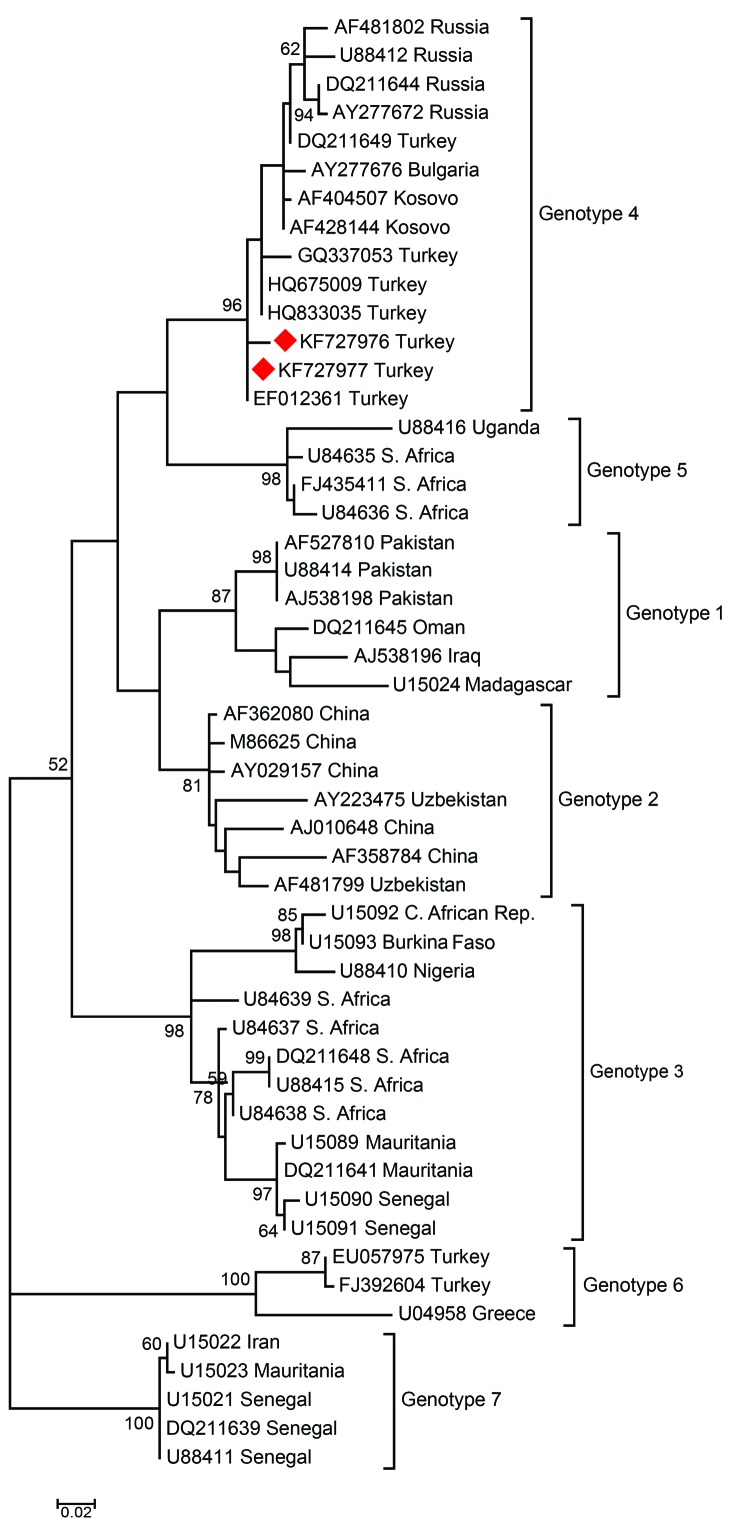
Phylogenetic tree of nucleotide sequences of CCHFV. Phylogenetic tree based on the 260 bp of the small segment of the CCHFV genome. The multiple sequence alignment was obtained by using MEGA 5.1 (http://www.megasoftware.net), and the phylogenetic tree was constructed by the maximum-likelihood method using 1,000 bootstrap replicates of the sequence data. The tree is drawn to scale with branch length in the same unit as those of the evolutionary distance used to infer the phylogenetic tree. The phylogenetic tree includes the 7 genotypes described by Mild et al. (*8*). Bootstrap confidence limits (>50) are shown at each node. The geographic origin is given for each sequence. The CCHFV Samsun Turkey described in this report is shown by the diamond. Scale bar indicates number of nucleotide substitutions per site. CCHFV, Crimean-Congo hemorrhagic fever; S., South; C., Central.

The Samsun CCHFV partial sequences of the S segments obtained in this study have been deposited in GenBank under the accession nos. KF727976 and KF727977. CCHFV are distributed within 7 different genotypes in the world. The CCHFV sequences obtained in the present study belong to genotype 4 (Figure 1).

## Conclusions

**Figure 2:**
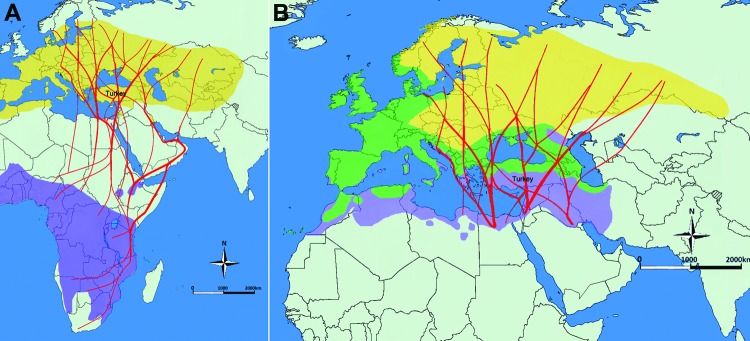
Migration patterns of birds carrying ticks with Crimean-Congo hemorrhagic fever virus. A) Great reed warbler (*Acrocephalus arundinaceus*) eastern migration routes (red lines), breeding grounds (yellow) and wintering areas (purple). Bodies of water are blue, and nonbreeding/nonwintering areas are light green.. B) European robin (*Erithacus rubecula*) eastern migration routes (red lines), resident grounds (green), breeding grounds (yellow), and wintering areas (purple). Bodies of water are blue, and nonbreeding/nonwintering areas are light green.

We detected CCHFV RNA in *Hyalomma* spp. (nymphs) collected on great reed warblers and in *Ixodes* spp. (nymphs) on European robins, which migrate across Turkey twice a year en route from their breeding sites to their wintering sites (Figure 2) and back. The probability of CCHFV transportation by ticks among different regions and countries is high during migration of both bird species. Because these birds stop several times during migration (*9*), CCHF in Europe possibly could increase, especially at the stopover sites in southern Europe, which provide suitable ecologic environments.

Although *Hyalomma* ticks are the most commonly encountered ticks that carry CCHFV in Turkey, the virus also was detected in ixodid ticks, such as *Rhipicephalus* spp. and *Haemaphysalis* spp. picked up from humans and animals (*10*). Also, Albayrak et al. (*11*) detected CCHFV in *I. ricinus* ticks. Because the 2 sequences detected showed similarity with CCHFV genotype 4, which was widespread in Turkey (*1*), whether the ticks were infected in Turkey or infected earlier during bird migration is impossible to say. Other studies have shown that CCHFV could be transported by ticks on birds (*12*–*14*). By itself, transportation of infected ticks by birds might not be sufficient to cause the epidemics in Turkey, but along with this, climate changes, environmental changes, increased number of sensitive animals, and tick and animal movements might play a role in spreading CCHF (*1*).

In ecologically important regions, such as the Kizilirmak Delta, where resident and migratory birds are mixed, different microorganisms or ticks can be transferred among birds, and then carried by birds for long distances. Therefore, knowing migration routes and what pathogens birds are infected with may help predict future epidemics in various countries and provide highlight the geographic regions where diseases could be detected (*15*).

## References

[1] Leblebicioglu H. Crimean-Congo haemorrhagic fever in Eurasia. Int J Antimicrob Agents. 2010;36(Suppl 1):S43–6 and. 10.1016/j.ijantimicag.2010.06.020 20810253

[2] Uyar Y, Christova I, Papa A. Current situation of Crimean Congo hemorrhagic fever (CCHF) in Anatolia and Balkan Peninsula. Turk Hij Deney Biyol Derg. 2011;68:139–51. 10.5505/TurkHijyen.2011.60352

[3] Shirihai H, Kirwan GM, Yosef R. Raptor migration in Israel and the Middle East: a summary of 30 years of field research. Eilat (Israel): International Birding & Research Center in Eliat; 2000.

[4] Bariş S, Erciyas K, Gürsoy A, Özsemir C, Nowakowski J. Cernek—a new bird ringing station in Turkey. Ring. 2005;27:113–20.

[5] Walker AR, Bouattour A, Camicas J-L, Estrada-Peña A, Horak IG, Latif AA, Ticks of domestic animals in Africa: a guide to identification of species. Edinburgh (Scotland): Bioscience Reports; 2003.

[6] Yapar M, Aydogan H, Pahsa A, Besirbellioglu BA, Bodur H, Basustaoglu AC, Rapid and quantitative detection of Crimean-Congo hemorrhagic fever virus by one-step real-time reverse transcriptase–PCR. Jpn J Infect Dis. 2005;58:358–62 . 16377867

[7] Schwarz TF, Nsanze H, Longson M, Nitschko H, Gilch S, Shurie H, Polymerase chain reaction for diagnosis and identification of distinct variants of Crimean-Congo hemorrhagic fever virus in the United Arab Emirates. Am J Trop Med Hyg. 1996;55:190–6 . 8780459 10.4269/ajtmh.1996.55.190

[8] Mild M, Simon M, Albert J, Mirazimi A. Towards an understanding of the migration of Crimean-Congo hemorrhagic fever virus. J Gen Virol. 2010;91:199–207 and. 10.1099/vir.0.014878-0 19812264

[9] Alerstam T, Christie DA. Bird migration. Cambridge (UK): Cambridge University Press; 1993.

[10] Tekin S, Bursali A, Mutluay N, Keskin A, Dundar E. Crimean-Congo hemorrhagic fever virus in various ixodid tick species from a highly endemic area. Vet Parasitol. 2012;186:546–52 and. 10.1016/j.vetpar.2011.11.010 22119389

[11] Albayrak H, Ozan E, Kurt M. Molecular detection of Crimean-Congo haemorrhagic fever virus (CCHFV) but not West Nile virus (WNV) in hard ticks from provinces in northern Turkey. Zoonoses Public Health. 2010;57:e156–60 and. 10.1111/j.1863-2378.2009.01316.x 20163578

[12] Zeller HG, Cornet JP, Camicas JL. Crimean-Congo haemorrhagic fever virus infection in birds: field investigations in Senegal. Res Virol. 1994;145:105–9 and. 10.1016/S0923-2516(07)80012-4 8059064

[13] Lindeborg M, Barboutis C, Ehrenborg C, Fransson T, Jaenson TG, Lindgren PE, Migratory birds, ticks, and Crimean-Congo hemorrhagic fever virus. Emerg Infect Dis. 2012;18:2095–7 and. 10.3201/eid1812.120718 23171591 PMC3557898

[14] Palomar AM, Portillo A, Santibanez P, Mazuelas D, Arizaga J, Crespo A, Crimean-Congo hemorrhagic fever virus in ticks from migratory birds, Morocco. Emerg Infect Dis. 2013;19:260–3 and. 10.3201/eid1902.121193 23347801 PMC3559059

[15] Tsiodras S, Kelesidis T, Kelesidis I, Bauchinger U, Falagas ME. Human infections associated with wild birds. J Infect. 2008;56:83–98 and. 10.1016/j.jinf.2007.11.001 18096237 PMC7172416

